# Field effectiveness and safety of fluralaner plus moxidectin (Bravecto® Plus) against ticks and fleas: a European randomized, blinded, multicenter field study in naturally-infested client-owned cats

**DOI:** 10.1186/s13071-018-3175-z

**Published:** 2018-11-19

**Authors:** Nadja Rohdich, Eva Zschiesche, Oliver Wolf, Wolfgang Loehlein, Thierry Pobel, Maria José Gil, Rainer K. A. Roepke

**Affiliations:** 1 MSD Animal Health Innovation GmbH, Zur Propstei, 55270 Schwabenheim, Germany; 2 Loehlein & Wolf Vet Research, Maistrasse 69, 80337 Munich, Germany; 3 TPC Biomed, C/Los Betetas 12-4°D, 42002 Soria, Spain

**Keywords:** Bravecto Plus, Ectoparasites, Feline, Fipronil, Fleas, Fluralaner, Isoxazoline, Moxidectin, *Rhipicephalus*, Ticks

## Abstract

**Background:**

A spot-on formulation containing fluralaner (280 mg/ml) plus moxidectin (14 mg/ml) (Bravecto® Plus) has been developed to provide broad spectrum parasite protection for cats. The effectiveness and safety of this product against ticks and fleas was assessed in a randomized, controlled, 12-week study in client-owned cats in Germany and Spain.

**Methods:**

Eligible households containing at least one cat with at least two fleas and/or two ticks were allocated randomly in a 2:1 ratio to a single treatment with fluralaner plus moxidectin on Day 0, or three 4-weekly treatments with fipronil (Frontline®). Veterinary staff, masked to treatment, completed tick and flea counts on each cat at 14 ± 2 (2 weeks), 28 ± 2 (4 weeks), 56 ± 2 (8 weeks) and 84 ± 2 days (12 weeks) after the initial treatment.

**Results:**

In total, 707 cats (257 with ticks) from 332 households (236 with fleas) were included. *Ixodes ricinus* (78%) and *Rhipicephalus sanguineus* complex (18%) ticks were the most commonly identified. Tick and flea counts were lower in the fluralaner plus moxidectin group than in the fipronil group throughout the study and the efficacy of fluralaner plus moxidectin exceeded 97 and 98%, respectively. At 12 weeks, 94.1 and 93.3% of cats from the fluralaner plus moxidectin and 92.2 and 60.3% of cats from the fipronil group were free of ticks and fleas, respectively. Fluralaner plus moxidectin was non-inferior to fipronil (*P* < 0.0001) at all assessments and superior to fipronil at 2 and 8 weeks for the proportion of cats free of ticks (*P* < 0.0001). Fluralaner plus moxidectin was superior to fipronil for the proportion of both households and cats free of fleas (*P* < 0.0001). Both products were safe and well tolerated.

**Conclusions:**

A single application of fluralaner plus moxidectin spot-on was well tolerated by cats and highly effective for 12 weeks against ticks and fleas. Fluralaner plus moxidectin was non-inferior to fipronil for the proportion of ectoparasite-free and consistently superior to fipronil in controlling fleas.

## Background

A key part of veterinary preventive healthcare in cats is the treatment and/or prevention of ecto- and endoparasite infestations. The prevalence of flea infestations in cats is generally higher than that of ticks [1]. However, there is a dearth of information on feline tick infestations. The most common genera of ticks that are found on cats are *Ixodes* spp. and *Rhipicephalus* spp. [2, 3], but the overall prevalence of tick infestations is likely underestimated since they may go unnoticed, unless attached to prominent sites on a cat’s head, or be removed by grooming behaviour. A recent survey in Austria, Belgium, France, Hungary, Italy, Romania and Spain confirmed tick and/or flea infestation in 16.7% of 1519 client-owned cats [1]. Interestingly, co-infection with gastrointestinal nematodes (most commonly *Toxocara cati*) was found to be common (11.9%).

The modern era of ectoparasite control for cats began in the mid-1990s with the advent of low-volume, monthly-applied topical products. The earliest of these products, fipronil (effective against fleas, with some tick efficacy) and imidacloprid (fleas only), were more convenient in terms of formulation (spot-on, compared to sprays, dusts and bathing) and safer than earlier flea control products (e.g. organophosphates) [4]. The early 2000’s saw the introduction of selamectin, a topically applied but systemically acting macrocyclic lactone. Despite lacking tick efficacy in cats, topically applied selamectin provided owners with improved convenience because of its extended spectrum of activity beyond fleas and ear mites (*Otodectes cynotis*) to include treatment of adult intestinal roundworms and intestinal hookworms and prevention of heartworm disease [5]. In 2009, a monthly spot-on combination product containing imidacloprid plus the systemically active macrocyclic lactone moxidectin was registered for use in cats with a similar spectrum of activity as selamectin [6, 7]. More recently, a monthly spot-on product introduced for cats combined fipronil with the insect growth regulator (*S*)-methoprene, the anticestodal agent praziquantel and the macrocyclic lactone eprinomectin to provide efficacy against fleas, ticks, gastrointestinal nematodes, lungworms and tapeworms, and prevention of heartworm disease [8]. In 2017, a combination of selamectin and the isoxazoline sarolaner, both with a systemic mode of action, was commercialized in Europe as a monthly spot-on for cats, extending the spectrum of the selamectin product to include ticks [9]. Thus there has been a substantial evolution in the convenience and spectrum of activity of topically applied products available for cat owners.

Nonetheless, despite these advances, a potential limitation of these products lies in the need for repeated monthly applications. This is important in light of a recent survey in Europe showing that cats treated less than four times per year with monthly products are at a significantly greater risk of flea infestation than those treated more frequently [1]. Similarly, the control of endoparasites is dependent on owner compliance, such as the minimum of four treatments per annum proposed by the European Scientific Council Companion Animal Parasites [10]. Despite this expert guidance, ensuring pet owner compliance with control measures for internal and external parasites in cats and dogs continues to present a substantial challenge for the veterinary profession [11–14]. Therefore there is an ongoing need for products with the potential to improve owner compliance with veterinary treatment recommendations.

A spot-on formulation of fluralaner, an extended-duration isoxazoline compound with potent insecticidal and acaracidal activity in these species, was introduced to help address that need. Clinical studies in client-owned dogs and cats have confirmed the safety and effectiveness of fluralaner in providing up to 12 weeks control of flea and tick infestations under field conditions [15–19]. While there are no reports of methods that would facilitate improved cat owner compliance with veterinary-recommended parasite control programs, a study of dog owners found that fluralaner’s sustained activity could lead to improved compliance with such programs [14].

In order to provide a broader spectrum of activity in a low volume spot-on formulation, fluralaner was combined with moxidectin, a well-known safe and effective macrocylic lactone with potent nematocidal activity, long half-life and safety profile that have enabled its use in extended duration formulations in dogs [20]. Moxidectin has been used in cats for more than 15 years in a monthly spot-on product at a dose rate of 1 mg/kg. This novel spot-on solution containing fluralaner (minimum recommended dose rate of 40 mg/kg) plus moxidectin (minimum recommended dose rate of 2 mg/kg), is now approved for cats for the treatment and control of tick and flea infestations for 12 weeks, for the prevention of heartworm disease for 8 weeks, and the treatment of nematode infections. A European field study demonstrated the effectiveness and safety of this product in the treatment of natural infections with gastrointestinal parasites (roundworms and hookworms) and *Capillaria* spp. in client-owned cats [21]. The present study reports the effectiveness and safety of this combination product in the treatment and control of natural tick and flea infestations of client-owned cats.

## Methods

### Study design

This was a multicenter, positive-controlled, randomized, investigator-blinded study, conducted from March until October 2015, in 33 veterinary practices located in Germany and Spain. The study was conducted in consideration of Good Clinical Practice VICH guideline GL9, EMEA, 2000, Guideline on Statistical Principles for Veterinary Clinical Trials (EMEA, 2010), Guideline for the testing and evaluation of the efficacy of antiparasitic substances for the treatment and prevention of tick and flea infestation in dogs and cats (EMEA/CVMP/EWP/005/2000-Rev.2) and the World Association for the Advancement of Veterinary Parasitology (WAAVP) guidelines for evaluating the efficacy of parasiticides for the treatment, prevention and control of tick and flea infestation on dogs and cats [22–25]. Cat owners completed an informed consent form for the inclusion of all cats in a household into the study prior to any enrollment and prior to initiation of treatment.

### Animals and households

Healthy cats at least 10 weeks-old and weighing at least 1.2 kg were eligible for inclusion. Cats with chronic medical conditions could be included at the discretion of the investigator in each clinic. Households were eligible for enrollment if they contained at least one cat with at least two fleas and/or at least two ticks, and were excluded if they contained a pregnant or lactating cat, if more than five cats were present, or if they contained non-feline animals capable of hosting fleas or ticks. All cats in each enrolling household received the same treatment.

To be eligible for enrollment, cats could not have received ectoparasiticide treatment within the previous 7 to 30 days, depending on the expected duration of effect of the treatment. No household environmental flea treatment was allowed for two months before the start of the study. During the study, the use of any non-study products with insecticidal or insect growth regulator properties was not permitted on either pets or premises of participating households. Grooming and bathing were allowed during the study, but should not have been performed for three days before a scheduled visit or for three days after treatment.

In the European Union, the guideline for the demonstration of efficacy against ticks and fleas requires 50 treated cases per region in two geographical regions for each of ticks and fleas, meaning a total of 150 cats infested with ticks would be included in the study (100 cats in the fluralaner plus moxidectin group and 50 in the fipronil group) [24]. Assuming a drop-out rate of 15% and an average of two tick-infested cats per household, 90 households with 180 tick-infested cats were to be included. A similar calculation for households with flea-infested cats provided the same household enrollment requirement. It was assumed that 50% of enrolling households with a tick-infested cat would have at least one flea-infested cat, so that the total number of households to be enrolled was 225 (based on the resulting assumption of 45 households with ticks only, 45 households with ticks and fleas and 135 with fleas only).

Owners were instructed to record any between-visit observations related to tick or flea infestation and to ensure that ticks were collected and brought to the practice within one week of observation, or to immediately arrange for an additional visit. Collected ticks were shipped to a central laboratory in Germany (IDEXX laboratories, Ludwigsburg) for identification to the genus and species level. If lesions of flea allergy dermatitis were present, the size, type (erythema, papules, crusts, scales, alopecia, excoriation) and localization of the largest lesion were also documented. Clinic staff administering treatment were not blinded; all clinic staff involved in study assessments were masked to treatment.

### Randomization and treatment

Using computer-generated randomization lists, households were randomly allocated to treatment groups stratified by site in blocks of three, in a 2:1 ratio for the fluralaner plus moxidectin spot-on to a commercially-available fipronil spot-on product. All treatments were administered within each clinic, by clinic staff.

The fluralaner (280 mg/ml) plus moxidectin (14 mg/ml) product (Bravecto® Plus spot-on for cats) was supplied in pipettes containing 0.4, 0.89 and 1.79 ml for cats of 1.2–2.8 kg, > 2.8–6.25 kg and > 6.25–12.5 kg body weight, respectively. Treatment was applied topically on a single occasion, Day 0, at a dose rate of 40–94 mg fluralaner plus 2.0–4.65 mg moxidectin/kg body weight. For application, the cat was required to be standing or lying in sternal recumbency with its back horizontal. Treatment was applied by placing the tip of the pipette on the skin at the base of the cat’s skull and then gently squeezing to apply the entire contents directly onto the cat’s skin. The potential for product run-off was minimized by limiting the amount applied to any one spot: if two spots were needed, the first was applied at the base of the skull and the second between the shoulder blades.

Fipronil (Frontline® spot-on cat 10% w/v solution, Boehringer Ingelheim, Ingelheim, Germany) was supplied in pipettes containing 0.5 ml for cats weighing at least 1 kg body weight. Treatment was applied topically on Days 0, 28 ± 2 and 56 ± 2, based on the minimum treatment interval for the product of 4 weeks, at a dose rate of approximately 7.5–15 mg/kg body weight. The product was applied as spots along the back: one at the base of the skull and a second, if needed, 2 to 3 cm distal to this, according to manufacturer’s instructions. Care was taken to apply the product directly to the skin and to avoid excessive wetting of the hair at the treatment spot, as the manufacturer reports that it causes a sticky appearance for up to 24 h after application.

After treatment, each cat was inspected to determine if there had been any product run-off. Cats were observed for 10 min to determine if any skin irritation was present at the application site. Owners were instructed to observe their cats for any adverse events (i.e. unfavorable or unexpected events), and to contact the investigator to report any such events immediately after they were observed.

### Assessments

On Day 0, each cat was thoroughly examined by the investigator to determine general health and suitability for inclusion in the study. At this and all scheduled subsequent visits after 14 ± 2 (2 weeks), 28 ± 2 (4 weeks), 56 ± 2 (8 weeks) and 84 ± 2 days (12 weeks), physical examinations were completed, ticks and fleas were counted, ticks were collected for identification and signs of flea allergy dermatitis assessed. Safety assessments were based on all observations of adverse events by owners or clinic staff in all cats enrolled and allocated to a treatment group [intention-to-treat (ITT) population].

Tick and flea counts were performed by trained clinic staff using the comb-counting method described in WAAVP guidelines for evaluating the efficacy of parasiticides for the treatment, prevention and control of tick and flea infestations on cats [25]. If required, cats could be sedated immediately prior to combing. Assessment of tick infestations involved pushing against the natural lay of the hair to expose any fleas or ticks, whether or not attached. All ticks were gently removed using forceps, counted and classified as live or dead. Assessments continued in this manner for at least 5 min. After this assessment was completed, cats were combed from front (including the whole head, ears and neck) to back (including the tail, flanks, legs, chest, axillae, groin, ventral thorax and abdomen) using overlapping strokes, for at least 5 min with a fine-toothed flea comb (approximately 11–13 teeth/cm). Special attention was paid to ectoparasite predilection sites (in hair whorls beneath the ears and hind legs, axillae and ventral abdomen, tail-base and back just cranial to the tail). If ticks and/or fleas were recovered during combing, the procedure was continued for a further 5 min until no ticks or fleas were recovered, making the total assessment time at least 10 min per cat. Between visits, owners were instructed to observe their cats for the presence of any live ticks and/or fleas and record the numbers. Any attached ticks that were observed between visits were to be removed with forceps and placed in clinic-supplied tubes labeled with the cat’s name, and taken to the clinic within one week for classification and identification. In the event that tick removal by the owner was not possible, the cat was to be brought to the clinic for an unscheduled visit.

### Statistical analysis

The primary endpoint of the study assessed all cats that were treated and examined according to the protocol [per protocol (PP) population]. The primary efficacy criterion was the percent reduction in tick and flea counts for each product at each follow-up visit, in comparison to the initial tick and flea burden. The statistical unit was the individual animal for tick efficacy and the household for flea efficacy. Efficacy analyses were also completed for the ITT population.

Study group means were determined for each visit (pre-treatment on Day 0 and follow-up visits at 14 ± 2, 28 ± 2, 56 ± 2 and 84 ± 2 days). The calculation was based on live ticks and fleas, in cats initially infested with ticks and in flea-infested households, respectively. The percent reduction in geometric and arithmetic mean counts was calculated for each study group and each follow-up visit according to the formula:

$$ \mathrm{Reduction}\ \left(\%\right)=\left(\overline{\mathrm{X}}_{\mathrm{pre}}-\overline{\mathrm{X}}_{\mathrm{post}}/\overline{\mathrm{X}}_{\mathrm{pre}}\right)\times 100 $$

where $\overline{\mathrm{X}}_{\mathrm{pre}}$ represents the mean of live ticks or fleas on Day 0, and $\overline{\mathrm{X}}_{\mathrm{post}}$ is the mean at each post-Day 0 assessment. To allow the calculation in case of zero counts, the geometric mean was calculated as follows:

$$ {\mathrm{x}}_{\mathrm{g}}={\left(\prod \limits_{\mathrm{i}=1}^{\mathrm{n}}\left({\mathrm{x}}_{\mathrm{i}}+1\right)\right)}^{\frac{1}{\mathrm{n}}}-1 $$

To compensate for the skewed distribution of geometric means, the tick or flea counts were log-transformed prior to statistical analysis: x_i_’ = ln (x_i_ + 1). Tick and flea counts at follow-up visits were compared pairwise to the pre-treatment counts using a one-sided, two-sample t-test. The level of significance (α) was set at 0.025.

Secondary efficacy was based upon the percentage of cats free of live ticks and/or fleas and households free of fleas. For each post-treatment follow-up visit, non-inferiority and superiority of the percentage of tick or flea-free cats in the fluralaner plus moxidectin group were compared to the percentage of tick or flea-free cats in the fipronil group. A test of non-inferiority for the risk difference was used with an α of 0.025 and a tolerated difference (δ) of 0.15 [26]. The *P*-value and the lower 97.5% one-sided confidence limits were calculated. If the lower confidence limit was above -0.15, it was concluded that fluralaner plus moxidectin was no less effective (non-inferior) to fipronil. If the lower confidence limit was above 0, it was concluded that fluralaner plus moxidectin was superior to fipronil.

Frequency tables were used to compare the distribution of sex, breed, hair length, living conditions, number of cats in the household and presence of skin lesions possibly related to flea allergy dermatitis in both treatment groups. The presence of clinical signs of flea allergy dermatitis and improvement in those signs were evaluated descriptively.

## Results

In total, 332 households with at least one cat qualified for enrollment. The targets for inclusion of tick-infested cats (*n* = 50) and flea-infested households (*n* = 50) were met in both Germany and Spain. For ticks, the PP population included 229 cats (136 in Germany, 93 in Spain) and the ITT population 257 cats (154 in Gemany, 103 in Spain). For fleas, the PP population included 208 households with at least one flea-infested cat (88 in Gemany, 120 in Spain) and the ITT population 236 households (103 in Gemany, 133 in Spain). There were 707 cats involved in the ITT population and 635 cats in the PP population. Initial homogeneity between study groups was demonstrated at inclusion (Day 0) for cats from all households. There was more than one cat in approximately 60% of households in each group, 14 and 16% of cats in the fluralaner plus moxidectin and fipronil groups, respectively, were reported as inside cats, and 79 and 73% of cats, respectively, were reported by owners to spend time both inside and outside. The breed distribution was similar between groups and included European (*n* = 380), mixed breed (*n* = 28), Persian (*n* = 22) and Siamese (*n* = 14) cats, with low numbers of British shorthair (*n* = 5), Maine Coone (*n* = 4), Birman (*n* = 3), Ragdoll (*n* = 2), Tonkinese (*n* = 1), Turkish Angora (*n* = 1), Havana (*n* = 1) and Chartreux (*n* = 1). For the ITT population the average age was 4.9 years in the fluralaner plus moxidectin group and 4.8 years in the fipronil group. The mean weights were 4.2 and 4.1 kg, respectively. Males comprised 57% of cats in the fluralaner plus moxidectin group and 52% of cats in the fipronil group, and 83% of cats in each group had been neutered.

At enrollment, six cats with concomitant disease (epilepsy, hyperthyroidism, hypertension, congestive heart failure, feline leukemia virus infection) requiring long-term treatment (phenobarbital, carbimazole or thiamazole, amlodipine, benazepril and interferon-alpha, respectively) were included in the fluralaner plus moxidectin group. A single cat in the fipronil group was stabilized at enrollment on benazepril and furosemide for congestive heart failure, and this was continued during the study.

During the study 72 cats were either withdrawn, lost to follow up, or excluded from a visit analysis: 35 cats were excluded from the efficacy analysis because of protocol violations, mainly due to being washed or groomed within the proscribed pre- or post-treatment interval, or for failure to adhere to the scheduled visits; data from 17 fluralaner plus moxidectin group cats were excluded from the efficacy analysis (but were included in the safety analysis) because the incorrect pipette was used, meaning that the applied dose rate exceeded the maximum recommended; 13 cats were lost to follow-up; 5 cats in the fluralaner plus moxidectin group died [two road traffic accidents, two with no further details were available (one accidentally, one found dead) and one was euthanized due to weight loss, lymphadenopathy and dyspnea (attributed to a malignant lymphoma)]. None of these deaths were attributable to treatment. One cat from the fluralaner plus moxidectin group was withdrawn by the owner due to a reported lack of efficacy, and one cat from the fipronil group cat was withdrawn because of a reported intolerance to the product.

A total of 873 ticks (ITT population) were collected at inclusion: the most frequent tick species found was *Ixodes ricinus* (*n* = 684, 78.4%, 1–57 per cat) in both Germany and Spain; *Rhipicephalus sanguineus* complex (*n* = 154, 17.6%, 1–4 per cat) mainly in Spain (two cats in Germany were infested); and *Dermacentor reticulatus* (*n* = 2, 0.2%, 1 per cat), *Dermacentor marginatus* (*n* = 2, 0.2%, 1 per cat), *Haemaphysalis concinna* (*n* = 2, 0.2%, 2 ticks per cat) and *Ixodes* spp. (*n* = 1, 0.1%) were also found, as well as *Ixodes* spp. larvae (*n* = 15, 1.7%, 1–4 per cat) and nymphs (*n* = 13, 1.5%, 1–2 per cat) in Spain.

**Fig. 1:**
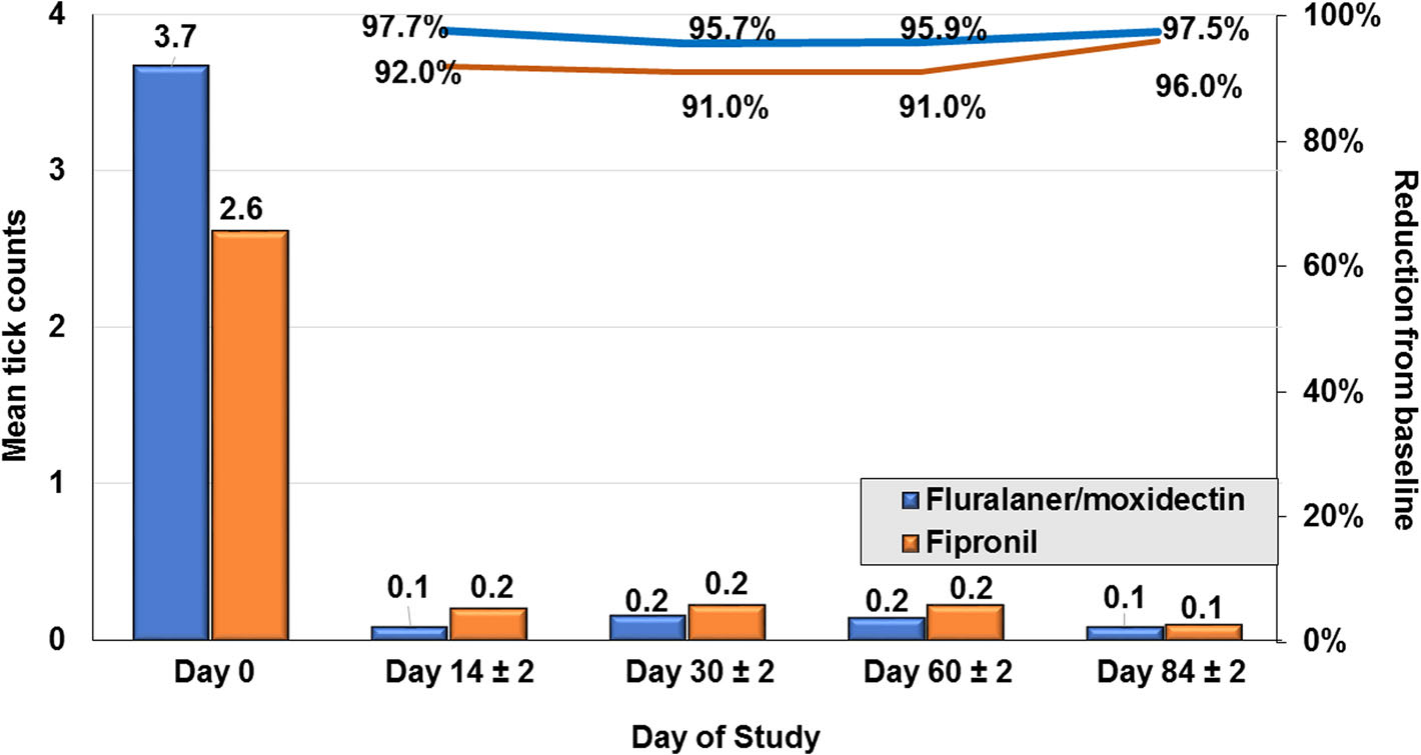
Arithmetic mean tick counts and percent reduction from baseline at each subsequent visit for topical fluralaner plus moxidectin- or fipronil-treated cats (bars indicate arithmetic mean tick counts; lines indicate percent reductions from baseline)

**Fig. 2:**
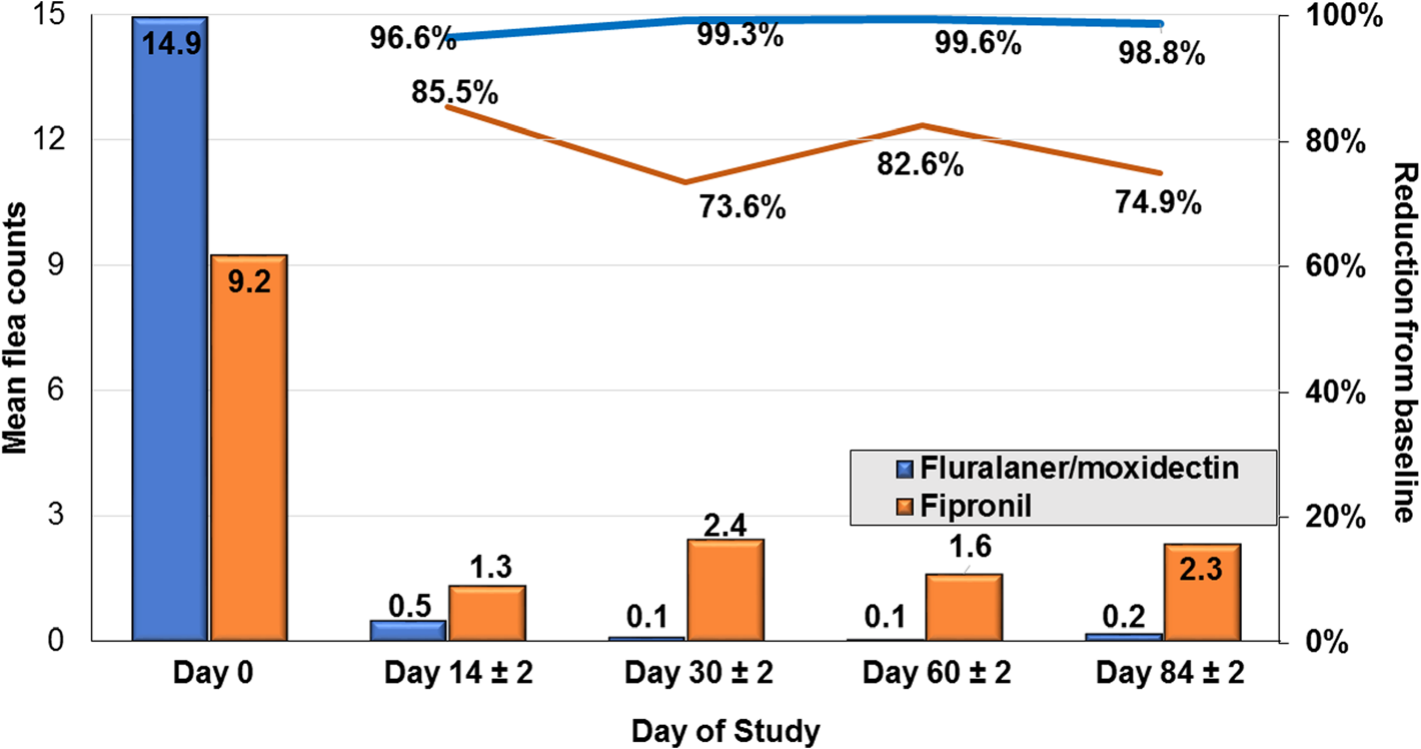
Arithmetic mean flea counts and percent reduction from baseline at each subsequent visit for topical fluralaner plus moxidectin- or fipronil-treated cats (bars indicate arithmetic mean flea counts; lines indicate percent reductions from baseline)

At each follow-up assessment, mean tick and flea count reductions in both groups were significant relative to Day 0 (Tables 1, 2; Figs. 1, 2). The mean tick and flea count reductions from baseline in the fluralaner plus moxidectin group were greater than in the fipronil group at all post-Day 0 assessments. For the PP population at 2, 4, 8 and 12 weeks, the geometric mean live tick count reductions in the fluralaner plus moxidectin group were at least 97.2%, and in the fipronil group were at least 92.7%. For the PP population at 2, 4, 8 and 12 weeks, the geometric mean flea count reductions in the fluralaner plus moxidectin group were at least 98.9% (arithmetic means at least 96.6%) while in the fipronil group these reductions were at least 86.3% (arithmetic mean 74.9%), and exceeded 90% on only one occasion, two weeks following the first treatment (Table 2, Fig 2).

**Table 1:** Geometric (arithmetic) mean counts of live ticks and percent reduction from baseline in each group

Visit	Mean	Reduction (%)	*t-*statistic (*t*_*df*_)	*P*-value (Pr > t)	Mean	Reduction (%)	*t*-statistic (*t*_*df*_)	*P*-value (Pr > t)
Per protocol population								
	Fluralaner + moxidectin (*n* = 152)				Fipronil (*n* = 77)			
1	2.59 (3.67)	–			2.17 (2.61)	–		
2	0.05 (0.09)	98.3 (97.7)	*t*_(200.37)_ = 26.6	<0.0001	0.12 (0.21)	94.4 (92.0)	*t*_(152)_ = 17.9	<0.0001
3	0.07 (0.16)	97.2 (95.7)	*t*_(228.96)_ = 24.9	<0.0001	0.11 (0.23)	94.9 (91.0)	*t*_(152)_ = 17.6	<0.0001
4	0.07 (0.15)	97.3 (95.9)	*t*_(228.17)_ = 25.0	<0.0001	0.16 (0.23)	92.7 (91.0)	*t*_(144.27)_ = 17.5	<0.0001
5	0.05 (0.09)	97.9 (97.5)	*t*_(199.31)_ = 26.5	<0.0001	0.07 (0.10)	97.0 (96.0)	*t*_(119.53)_ = 21.0	<0.0001
Intent to treat population								
	Fluralaner + moxidectin (*n* = 171)				Fipronil (*n* = 86)			
1	2.54 (3.61)	–			2.22 (2.70)	–		
2	0.04 (0.08)	98.4 (97.9)	*t*_(219.73)_ = 28.1	<0.0001	0.17 (0.34)	92.2 (87.6)	*t*_(170)_ = 16.0	<0.0001
3	0.08 (0.15)	96.9 (95.6)	*t*_(255.62)_ = 25.9	<0.0001	0.15 (0.35)	93.2 (87.0)	*t*_(169)_ = 16.1	<0.0001
4	0.07 (0.14)	97.3 (96.0)	*t*_(252.87)_ = 26.2	<0.0001	0.15 (0.22)	93.4 (92.0)	*t*_(156.24)_ = 18.6	<0.0001
5	0.06 (0.10)	97.6 (97.1)	*t*_(230.92)_ = 27.2	<0.0001	0.07 (0.11)	96.9 (96.0)	*t*_(132.43)_ = 21.6	<0.0001

**Table 2:** Geometric (arithmetic) mean household flea counts and percent reduction from baseline

Visit	Mean	Reduction (%)	*t-*statistic (*t*_*df*_)	*P*-value (Pr > t)	Mean	Reduction (%)	*t-*statistic (*t*_*df*_)	*P*-value (Pr > t)
Per protocol population								
	Fluralaner + moxidectin (*n* = 135)				Fipronil (*n* = 73)			
1	6.89 (14.93)	–			6.38 (9.23)	–		
2	0.06 (0.51)	99.1 (96.6)	*t*_(186.21)_ = 24.9	<0.0001	0.50 (1.34)	92.1 (85.5)	*t*_(144)_ = 13.0	<0.0001
3	0.06 (0.10)	99.1 (99.3)	*t*_(154.39)_ = 26.4	<0.0001	0.71 (2.44)	88.8 (73.6)	*t*_(131.94)_ = 10.7	<0.0001
4	0.04 (0.06)	99.5 (99.6)	*t*_(145)_ = 27.2	<0.0001	0.66 (1.60)	89.7 (82.6)	*t*_(144)_ = 12.0	<0.0001
5	0.08* (0.18)	98.9 (98.8)	*t*_(168.67)_ = 25.6	<0.0001	0.87 (2.32)	86.3 (74.9)	*t*_(134.98)_ = 10.3	<0.0001
Intent to treat population								
	Fluralaner + moxidectin (*n* = 152)				Fipronil (*n* = 84)			
1	6.82 (14.26)	–			6.44 (10.0)	–		
2	0.08 (0.50)	98.9 (96.5)	*t*_(214.76)_ = 26.6	<0.0001	0.52 (1.46)	91.9 (85.4)	*t*_(166)_ = 13.4	<0.0001
3	0.06 (0.11)	99.1 (99.3)	*t*_(174.87)_ = 28.4	<0.0001	0.71 (2.49)	88.9 (75.1)	*t*_(156.73)_ = 11.2	<0.0001
4	0.03 (0.05)	99.5 (99.6)	*t*_(1628.1)_ = 29.4	<0.0001	0.61 (1.48)	90.5 (85.2)	*t*_(164)_ = 13.0	<0.0001
5	0.07 (0.16)	99.0 (98.9)	*t*_(189.22)_ = 27.7	<0.0001	0.81 (2.15)	87.4 (78.5)	*t*_(163)_ = 11.2	<0.0001

For secondary efficacy between-group comparisons, with the lower 97.5% one-sided confidence limit well above the non-inferiority limit of -0.15, fluralaner plus moxidectin non-inferiority to fipronil for tick and flea efficacy was shown (*P* < 0.0001) in the PP and ITT populations at each follow-up visit (Tables 3, 4). At all assessments following Day 0, the proportion of cats free of ticks was higher in the fluralaner plus moxidectin group than in the fipronil group. The fluralaner plus moxidectin treatment was superior to fipronil at 2 and 4 weeks for the number of cats free of ticks (*P* < 0.0001) and at 2, 4, 8 and 12 weeks for the proportion of households free of fleas and cats free of fleas (*P* < 0.0001). For the PP population at 2, 4, 8 and 12 weeks, at least 92.8 and 81.8% of tick-infested cats from the fluralaner plus moxidectin and fipronil groups, respectively, were free of ticks. For the PP population at 2, 4, 8 and 12 weeks, at least 93.3 and 60.3% of cats from the fluralaner plus moxidectin and fipronil groups, respectively, were free of fleas.

**Table 3:** Percent of cats infested with ticks on Day 0 that were free of ticks at subsequent visits

Treatment group	Cats free of ticks (%)			
	Visit 2	Visit 3	Visit 4	Visit 5
Per protocol population				
Fluralaner + moxidectin	96.1	92.8	93.4	94.1
Fipronil	88.3	89.6	81.8	92.2
Lower 97.5% one-sided confidence limit^a^	0.0008	-0.0456	0.0294	-0.0534
*P*-value for non-inferiority^b^	<0.0001	<0.0001	<0.0001	<0.0001
Intent to treat population				
Fluralaner + moxidectin	96.5	91.8	93.4	93.3
Fipronil	86.1	88.2	83.1	91.6
Lower 97.5% one-sided confidence limit^a^	0.0293	-0.0403	0.0215	-0.0530
*P*-value for non-inferiority^b^	<0.0001	<0.0001	<0.0001	<0.0001

^a^Lower 97.5% one-sided confidence limit was well above the non-inferiority limit of -0.15. If the lower confidence limit was above 0, superiority was concluded

^b^Farrington-Manning method

**Table 4:** Percent of households with at least one cat initially infested with at least two fleas that were free of fleas at subsequent visits

Treatment group	Households free of fleas (%)			
	Visit 2	Visit 3	Visit 4	Visit 5
Per protocol population				
Fluralaner + moxidectin	95.6	94.1	95.6	93.3
Fipronil	76.7	69.9	68.5	60.3
Lower 97.5% one-sided confidence limit^a^	0.0929	0.1388	0.1673	0.2190
*P*-value for non-inferiority^b^	<0.0001	<0.0001	<0.0001	<0.0001
Intent to treat population				
Fluralaner + moxidectin	94.7	93.4	96.0	93.9
Fipronil	75.0	70.2	69.5	61.7
Lower 97.5% one-sided confidence limit^a^	0.1046	0.1337	0.1682	0.2169
*P*-value for non-inferiority^b^	<0.0001	<0.0001	<0.0001	<0.0001

^a^Lower 97.5% one-sided confidence limit was well above the non-inferiority limit of -0.15. If the lower confidence limit was above -0.15, non-inferiority was concluded. If the lower confidence limit was above 0, superiority was concluded

^b^Farrington-Manning method

In the PP population there were 30 fluralaner plus moxidectin-treated cats (7.1%) and 6 fipronil-treated cats (2.8%) with clinical signs of flea allergy dermatitis at inclusion. Of these cats, in the fluralaner plus moxidectin group 86.7% had improved or were graded as clinically cured, compared with 66.7% in the fipronil group. Clinical cures were recorded in 53.3% of the fluralaner plus moxidectin cats and 33.3% of fipronil cats.

There were no treatment-related serious adverse events in either group. Adverse event reports in the fluralaner plus moxidectin group included a single report of itching at the site of application on Day 0; another cat was reported to show dyspnoea and was suspected to have been licking the application site the day after treatment; small spots of hair loss were reported in one cat on Day 4 and mild alopecia in a further eight cats, on a single occasion for each, between Days 13 through 15. In the fipronil group, alopecia at the application site was reported in two cats on Day 28; in a third cat crusting at the application site was observed on Day 35. On Day 0, salivation and lethargy in one cat from the fluralaner plus moxidectin group was considered to be possibly treatment-related. In the fipronil group, salivation and tremor observed in one cat on Day 0 was considered by the investigator to be probably treatment-related, as was itching, without further detail, observed in one cat on Day 29 and in two cats on Day 85. Isolated instances of mild, generally transient gastrointestinal signs considered unlikely to be treatment-related were reported to have occurred in both treatment groups at different times during the study.

## Discussion

To the best of our knowledge, this is the first reported field study demonstrating the 12-week field efficacy and safety of fluralaner against ticks in cats, and the first European field study confirming the 12-week efficacy and safety of fluralaner against fleas in cats. The new spot-on formulation of fluralaner plus moxidectin for cats (Bravecto® Plus) administered topically at 12-week intervals was safe and highly effective gainst natural tick and flea infestations in cats. The efficacy of fluralaner plus moxidectin was non-inferior to fipronil (*P* < 0.0001) at all time-points and superior to fipronil at two weeks and two months post-treatment for the proportion of cats free of ticks (*P* < 0.0001), and at all time-points for the proportion of households free of fleas and the proportion of cats free of fleas (*P* < 0.0001).

The numbers of ticks on fluralaner plus moxidectin-treated cats were reduced by at least 97.2% at all time-points after a single treatment. This tick efficacy in cats is consistent with that shown in a European field study (Germany, France and Spain) of fluralaner in dogs where tick counts were reduced by at least 99% at 2, 4, 8 and 12 weeks following a single treatment [15]. The results of the present study also compare favorably to those of two separate reports of isoxazolines described in European 12-week field studies in tick-infested, client-owned cats. In one study the efficacy against ticks of three consecutive monthly applications of a topical formulation of sarolaner and selamectin (an isoxazoline and macrocyclic lactone, respectively) was ≥ 92.6%, while the efficacy of fipronil administered according to the same schedule ranged from 74.6 to 93.4% [27]. The sarolaner-selamectin combination was non-inferior to fipronil at all time-points and superior on Days 30 and 60. In the other study, the efficacy of orally administered lotilaner against ticks ranged from 98.3 to 100%, and for fipronil from 89.6 to 99.6% [28]. Lotilaner was superior to fipronil from Days 14 to 70 and non-inferior on the other assessment days. The accumulated findings therefore indicate that while fipronil continues to be generally effective against ticks, it may be inferior to those isoxazolines against which it has been tested.

The results of this study in flea-infested households, a 98.9–99.5% reduction from baseline in geometric mean flea counts, provide evidence to support the immediate and sustained reduction in flea burdens for 12 weeks following a single fluralaner plus moxidectin treatment of cats. The results provide further substantiation of the efficacy of fluralaner against *Ctenocephalides felis*, which has been shown to be the dominant flea species in Europe [29]. These findings reinforce those from a USA field study where there was a 98.6–99.1% reduction in flea counts in treated cats for 12 weeks following a single fluralaner treatment [16]. In other studies with shorter-acting spot-on or oral products in cats, three consecutive monthly administrations have been required to reach 12 weeks of efficacy whereas fluralaner (with or without moxidectin) has been shown to achieve this efficacy duration following a single dose. The results of the present study compare favorably with those from two European (non-inferiority) field studies in cats, one investigating the flea control arising from three monthly applications of a combination of sarolaner and selamectin compared to three applications of a topical formulation of imidacloprid and moxidectin, the other comparing a single oral administration of lotilaner with a single application of fipronil/(S)-methoprene. In the former study, the three applications of sarolaner-selamectin resulted in mean flea count reductions from baseline of 97.3, 98.8 and 99.4% on Days 30, 60 and 90, respectively, and 83.6, 87.7 and 96.3% in the imidacloprid/moxidectin-treated group [27]. In the latter study, mean flea count reductions were 97.2 and 98.1% at two and four weeks post-treatment with lotilaner, respectively, while the corresponding efficacy for fipronil/(S)-methoprene was just 48.3 and 46.4% [30], respectively.

The low efficacy of fipronil in that study aligns with the findings in our study in which the fipronil group household mean flea count reductions were less than 90% on all but one occasion (2 weeks after the first treatment), and there was a low proportion of households (60.3%) that were free of fleas, despite the treatment being applied at the veterinary practice at 4-week intervals. While failures in the control of fleas on dogs and cats are common, they are frequently due to inappropriate control measures [30]. However, in the present study, fipronil treatment was applied every four weeks by the veterinary team. There is also considerable variation in the susceptibility of flea strains to insecticides [31, 32] and this may result in flea infestations that are difficult to control with certain agents under field conditions. It is clear from the results of our study and of other studies in Europe and the USA that fipronil, which in earlier papers had been shown to perform well under field conditions, often appears to perform poorly against fleas under the controlled conditions of a field study [16, 17, 30, 33–38].

While fleas are long established as important parasites of cats in Europe, concern about tick infestations in cats has received much less attention. Our finding of so many tick-infested cats, similar to that reported in 2017 by Geurden et al. [27], is an indicator that cats are at substantial risk from tick infestation, and therefore of the associated risk of infection with tick-borne pathogens. These recent findings suggest that more attention should be placed on the risks of tick infestations of cats, and of the potential such infestations have to result in vector-borne disease.

In the present study, in Germany and Spain, the predominant ticks prior to treatment were the sheep tick (*I. ricinus*, 78.4%) as well as other *Ixodes* spp. ticks (0.1%) including nymphs (1.5%) and larvae (1.7%) and the brown dog tick (*R. sanguineus* complex, 17.6%). Other *Ixodes* spp. found on cats, sometimes the predominate tick, can include the hedgehog tick (*I. hexagonus*) as reported in Belgium, France, Germany and Italy [2, 39, 40]. Both the sheep tick and brown dog tick also predominated in a study with sarolaner plus selamectin, although in that study *R. sanguineus* was found only on cats in France and Italy but not in Germany and Hungary. The same study reported low numbers of the ornate cow tick (*D. reticulatus*) on cats in Germany and Hungary and this was found in Spain in the present study along with low numbers of the ornate sheep tick (*D. marginatus*). The present study also found the relict tick (*H. concinna*), a common rodent tick, in low numbers on cats in Spain. This Eurasian hard tick has been previously reported in low numbers on dogs in Hungary [39, 40] but appears not to have been previously reported in cats. These findings underline that cats, through their behaviour can encounter questing ticks, meaning that a variety of ticks can be found.

Both immediate and persistent efficacy of ectoparasiticides are particularly important under field conditions where cats are exposed not only to re-infestation with ticks and fleas from the environment, but also to the risk of vector-borne pathogens that they carry. The extended-duration fluralaner plus moxidectin spot-on product tested in the present study was confirmed under field conditions to provide 12 weeks of activity following a single topical application. This will help to provide safe and effective extended duration ectoparasite control for cats in a form that reduces potential gaps in protection and is convenient to cat owners.

## Conclusions

The topical formulation of fluralaner plus moxidectin spot-on solution for cats was highly effective for 12 weeks against ticks [*I. ricinus*, *Ixodes* spp. (including nymphs and larvae), *R. sanguineus* complex, *D. reticulatus*, *D. marginatus*, *H. concinna*] and fleas (*Ctenocephalides* spp.) on naturally infested cats. It was safe and the percentage of parasite-free cases in the fluralaner-moxidectin group was higher and always significantly non-inferior to the registered fipronil spot on for cats.

## Abbreviations

EMEA: European Medicines Agency
FAD: Flea allergy dermatitis
ITT: Intent-to-treat
PP: Per protocol
VICH: International Cooperation on Harmonisation of Technical Requirements for Registration of Veterinary Medicinal Products
WAAVP: World Association for Veterinary Parasitology
X̅_pre_: Mean of live ticks or fleas on Day 0
X̅_post_: Mean at each post-Day 0 assessment
